# Micro‐ and Nano‐Bots for Infection Control

**DOI:** 10.1002/adma.202419155

**Published:** 2025-04-10

**Authors:** Azin Rashidy Ahmady, Shadman Khan, Hong Han, Wei Gao, Zeinab Hosseinidoust, Tohid F. Didar

**Affiliations:** ^1^ School of Biomedical Engineering McMaster University Hamilton Ontario L8S 4M1 Canada; ^2^ Andrew and Peggy Cherng Department of Medical Engineering Division of Engineering and Applied Science California Institute of Technology Pasadena CA 91125 USA; ^3^ Michael DeGroote Institute for Infectious Disease Research McMaster University Hamilton Ontario L8S 4L8 Canada; ^4^ Department of Chemical Engineering McMaster University Hamilton Ontario L8S 4L7 Canada; ^5^ Farncombe Family Digestive Health Research Institute McMaster University Hamilton Ontario L8S 4K1 Canada; ^6^ Department of Mechanical Engineering McMaster University Hamilton Ontario L8S 4L7 Canada

**Keywords:** anti‐biofilm machine, biosensor, infection, medical micro‐robot, nano‐robot

## Abstract

Medical micro‐ and nano‐bots (MMBs and MNBs) have attracted a lot of attention owing to their precise motion for accessing difficult‐to‐reach areas in the body. These emerging tools offer the promise of non‐invasive diagnostics and therapeutics for a wide range of ailments. Here, it is highlighted how MMBs and MNBs can revolutionize infection management. The latest applications of MMBs and MNBs are explored for infection prevention, including their use as accurate, minimally invasive surgeons and diagnosis, where they function as sensitive and rapid biosensors or carriers for contrast agents for real‐time imaging of infected tissue. Further, the applications are outlined in treatment serving as anti‐biofilm agents and smart carriers for antibiotics and anti‐infective biologics. The current challenges in designing MMBs and MNBs are highlighted for overcoming immune barriers, moving to deep infected tissue, and swimming in low Reynolds numbers and discuss mitigating strategies. Finally, as a future perspective, the potential advantages of multi‐drive propulsion, bioinspired, and artificial‐intelligence‐trained MMBs and MNBs are discussed, with a special focus on challenges and opportunities for their commercialization.

## Graphical abstract

This review presents a strategic vision for integrating micro‐ and nanobots in the pipeline for infection diagnosis, prevention, and treatment. To develop these robots as a practical solution for infection management, their design principles are clarified based on their propulsion mechanisms and then categorized infection management domains based on usage scenarios.

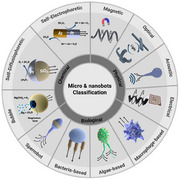

## 1 Introduction

Infection is the process of transmission and colonization of pathogenic organisms, including bacteria, viruses, and fungi, into the host's tissue, which results in a host response and could lead to severe health conditions.^[^ ^1^, ^2^ ^]^ In the case of bacteria, inappropriate administration of antibiotics for infection treatment has resulted in the emergence of antimicrobial‐resistant pathogens as a serious threat to human global health.^[^ ^3^, ^4^ ^]^ Viral, fungal, and parasitic infection management is also challenged by drug resistance and a lack of effective control strategies.^[^ ^5^, ^6^ ^]^ The World Health Organization (WHO) estimated the annual global mortality rate of antimicrobial‐resistant pathogens to be more than 4 million.^[^ ^6^ ^]^ Developing innovative measures for infectious disease management and reducing bacterial contamination are thus public health priorities.^[^ ^7^, ^8^, ^9^ ^]^ Infection management can be divided into various categories, including designing point‐of‐care biosensors intended for enabling early intervention or, even better, prevention of disease transmission,^[^ ^10^, ^11^ ^]^ developing biologics for treatment of infections caused by multi‐drug resistant microorganisms,^[^ ^12^, ^13^, ^14^ ^]^ expanding high‐resolution imaging systems for detection of infected tissues,^[^ ^15^, ^16^ ^]^ and advancing technologies for minimally invasive surgery that to mitigate risk of surgical site infection.^[^ ^17^, ^18^ ^]^ Micro‐ and nano‐bots are rapidly emerging as promising tools to address the many facets of infection management.^[^ ^19^, ^20^, ^21^ ^]^

Breakthrough advancements in various fields of science, including nanotechnology, materials sciences, artificial intelligence, and cell biology, have built a bridge between otherwise siloed disciplines and developed an interdisciplinary domain for designing miniature medical robots.^[^ ^22^, ^23^ ^]^ Medical micro‐bots (MMBs) and medical nano‐bots (MNBs) are referred to as the micro‐ and nano‐scale machines that exhibit spontaneous or externally controllable locomotion, which makes them suitable for a wide range of applications, including targeted cargo delivery, biosensing, detoxification, biopsy procedures, and miniature surgical interventions.^[^ ^24^, ^25^, ^26^ ^]^ In terms of their propulsion mechanism, MMBs and MNBs can be divided into three categories, namely chemically‐ driven, physically‐ driven, and biohybrid‐driven robots.^[^ ^27^ ^]^ These classes can be further classified into different subcategories. For instance, physical actuation robots can be magnetically‐,^[^ ^26^, ^28^ ^]^ optically‐,^[^ ^29^ ^]^ acoustically‐,^[^ ^30^ ^]^ or electrically‐^[^ ^31^ ^]^ driven, all of which are discussed in this review. MMBs and MNBs are also characterized based on their geometrical shape into two groups, namely shape‐persistent^[^ ^32^ ^]^ and shape‐morphing^[^ ^33^, ^34^ ^]^ bots, with the configuration being designed to match the application of choice.

Although MMBs and MNBs have significant potential in infection management and diagnostics owing to their small size, flexible structure, customizable propulsion mechanisms, and precise controllability,^[^ ^19^, ^21^, ^35^, ^36^ ^]^ challenges remain to be addressed regarding their stability and controllability in complex fluids, deep tissue penetration, and low Reynolds numbers flow.^[^ ^37^ ^]^

In this review, we present the current state of knowledge and notable contributions of MMBs and MNBs to the field of infection management. First, we outline of the fundamental propulsion mechanisms utilized in different micro‐ and nano‐robotic systems to optimize their control, efficiency, and functionality in diverse biomedical applications. Next, we categorize infection management domains based on usage scenarios for MMBs and MNBs into six different domains, namely applications in bio‐imaging and bio‐sensing of infections, as carriers of antimicrobials including small molecules and biologics, biofilm eradication, and minimally invasive surgical procedures. Finally, we examine current limitations of MMBs and MNBs for infection management in real‐life scenarios and propose strategies to optimize effectiveness.

## 2 Actuation and Drive of Medical Micro‐ and Nano‐Bots

**Figure 1:**
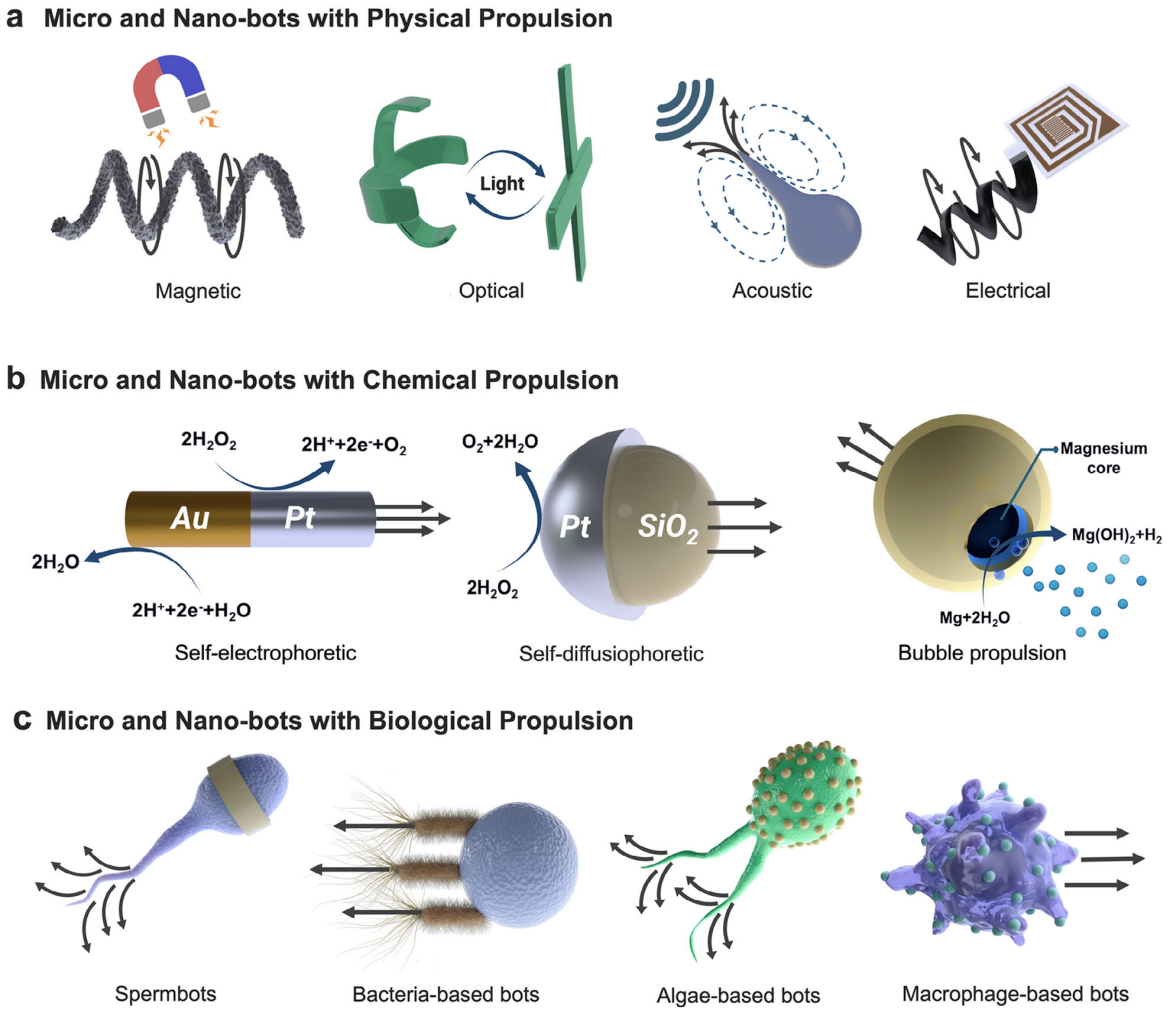
Classification of MMBs and MNBs based on their propulsion types. MMBs and MNBs are classified based on their propulsion mechanisms into three main groups, namely, physical, chemical, and biological‐based robots. a) Physical‐based robots are further divided into different subgroups, including magnetic, acoustic, optical, and electrical. b) Chemical and based MMBs and MNBs are subcategorized into self‐diffusiophoretic, self‐electrophoretic, and bubble propulsion. c) Biological‐based bots are subdivided into spermobots, algae‐based bots, bacteriobots, and macrophage‐based bots.

The idea of developing tiny machines that can swim between organs and whiting tissue performing functions such as surgery and cargo delivery has been a long‐held ambition of humans. Early studies for developing MMBs were mainly focused on designing their driving force, which resulted in the emerging wide range of micromotors.^[^ ^38^, ^39^ ^]^ This valuable body of knowledge serves as the basis of design for the next generation of miniaturized autonomous systems. Micro‐ and nano‐robotic systems are designed based on physical,^[^ ^40^ ^]^ chemical,^[^ ^41^, ^42^ ^]^ or biological^[^ ^43^ ^]^ propulsion, or a combination of two or more. The subcategories of each group, along with the pros and cons of each, are demonstrated in **Figure** **1** and summarized **Table** **1**.

**Table 1:** Classification of MMBs and MNBs based on their propulsion mechanism, with advantages and disadvantages of each group.

Type	Mechanism of propulsion	Advantages	Disadvantages	Refs.
Physical	Magnetic	Fast movement; Higher degrees of freedom for navigating them; Simple synthesis	The necessity of using Complicated and expensive navigator systems that prevent them from wide practical applications	[54, 63, 70, 71]
	Optical	Accurate spatial control; Simplicity of operation; Potential for enjoying real‐time optical tracking	Limited surface penetration of optical field that restricts their applications in deep tissues; generation of local heat	[40, 79, 81]
	Acoustic	Favorable biocompatibility; Ability for penetration in high depth; Simultaneous tracking capacities	Demanding precise and advanced studies for synthesizing them and their navigator systems	[83, 85, 87]
	Electrical	Feasibility for adjusting the speed and direction of motion with a simple operation; Potential for coupling them with artificial intelligence systems and developing smart medical robots	Demanding high voltage for operation in some cases; Sensitivity to electrical conductivity of biological media and other environmental factors	[50, 87, 95]
Chemical	Self‐ Electrophoretic	Simple structure; Needlessness for complicated navigator systems	Sensitivity to electrical conductivity of biological media; Low speed; Low controllability	[87, 104, 105]
	Self‐ Diffusuiophoretic	Able to move in solutions with high ionic strength; Needlessness for complicated navigator systems	Sensitivity to chemical gradient of biological media; Low speed; Low controllability	[102, 106]
	Bubble propulsion	High speed; Needlessness for complicated navigator systems	Demanding complicated design; Low controllability	[107, 109, 110]
Biological	Spermbot	Potential for application in gynecological drug delivery; Great biocompatibility	Low coupling efficiency for merging with other propulsion mechanisms	[117, 121]
	Bacteriobot	Potential for cancer treatment; High speed	Low biocompatibility; Short lifetime due to their elimination by the immune system; complicated culturing process	[124, 125, 126, 127, 128]
	Algae‐based bots	Able to move by multiple mechanisms; High speed; scalability	Low controllability in deep tissues	[130, 131, 135]
	Macrophage‐based bots	Great cargo‐loading; Effective delivery due to their engulfing function; compatible with the immune system; Penetration through biological barriers	Design complexity challenges due to their limited studies	[116, 137, 140]

### 2.1 Physical Propulsion

Physically powered MMBs and MNBs generally use external actuation sources such as magnetic‐^[^ ^44^, ^45^ ^]^ and electrical fields,^[^ ^46^ ^]^ optical‐^[^ ^47^ ^]^ and acoustic^[^ ^48^, ^49^ ^]^ waves, or a combination of those,^[^ ^50^, ^51^ ^]^ to provide fuel‐free and controllable locomotion.^[^ ^52^ ^]^ Physical propulsion offers distinct advantages, including enabling penetration in otherwise hard‐to‐reach sites of the body, rapid and long‐range locomotion, and high‐resolution navigation.^[^ ^53^ ^]^ However, applications could be significantly limited due to the requirement for an external source of energy and control systems. Preparing functional physical navigation systems as well as developing materials that respond to the physical propulsion mechanism of choice is a challenging procedure that restricts the options for designing physically driven MMBs and MNBs.^[^ ^54^ ^]^ Moreover, in many cases, working with and maintaining the external power sources requires the availability of specialized infrastructure and expertise,^[^ ^55^ ^]^ which restricts application even further.

#### 2.1.1 Micro‐ and Nano‐Bots Driven by Magnetic Actuation

Magnetic actuation has been studied and developed for over 3 decades and is among the most common modes of propulsion for bots.^[^ ^26^ ^]^ To design robots with magnetic actuation, the following principles should be considered: 1) Selecting proper magnetically responsive materials including ferromagnetic materials, for instance, iron,^[^ ^56^ ^]^ nickel,^[^ ^57^ ^]^ and cobalt,^[^ ^58^ ^]^ or paramagnetic materials, such as ferric oxide^[^ ^59^ ^]^; 2) optimizing fabrication method; 3) selecting appropriate magnetic actuation system.

Ferromagnetic materials possess unique electronic structures that generate a net dipole moment in response to an external magnetic field.^[^ ^60^ ^]^ Paramagnetic substances possess positive magnetic susceptibility because of the presence of unpaired electrons, though this is temporary and lower than ferromagnetic materials.^[^ ^61^, ^62^ ^]^ In other words, while both ferromagnetic and paramagnetic materials show a positive magnetic susceptibility (Xm), the order of magnitude in ferromagnets is considerably higher (Xm >> 0) than in paramagnetic materials (Xm > 0).^[^ ^52^ ^]^ Upon exposure to a low‐frequency external magnetic field, a magnetically responsive material exhibits magnetization properties that result in its motion. The velocity and direction of this motion can be controlled by optimizing the yaw angle and frequency of the rotating magnetic field.^[^ ^54^, ^63^ ^]^

Several fabrication methods have been developed for the preparation of MMBs and MNBs, such as stereolithographic 3D printing, mold casting, photopolymerization, electrospinning, and laser cutting. The choice usually depends on the final desired shape, size, and propulsion speed of the bot.^[^ ^64^, ^65^, ^66^ ^]^ Using these fabrication methods, MMBs and MNBs with a wide range of shapes can be produced, such as spherical, tubular**, **disk‐shaped, flagella, and rod‐shaped.^[^ ^62^ ^]^ Another important consideration here is the integration of magnetic materials into the robots’ structures, which can be achieved by either coating the MMB's structure with magnetic substances^[^ ^67^ ^]^ or embedding magnetic nanoparticles within an MMB's structure,^[^ ^68^ ^]^ and can be affected by the choice of fabrication method.

To generate the required external magnetic field electromagnetic sources, comprising Helmholtz and Maxwell coils, or permanent magnets that work based on translational motions or rotational mechanisms are utilized.^[^ ^54^ ^]^ Electromagnetic sources offer advantages such as modulating high‐frequency magnetic fields and controlling the generated field in a rapid manner and with high degrees of freedom based on the electric current of its coils.^[^ ^69^ ^]^ The downside is that electromagnets can be costly to implement in the context of biomedical applications, with energy efficiency and high temperature of coils being other concerns.^[^ ^70^ ^]^ In contrast, permanent magnets can function without generating heat because they do not require an external power supply.^[^ ^71^ ^]^ The main drawback here is the lack of freedom to adjust the magnetic field amplitude or to switch off the magnetic actuation system.^[^ ^72^ ^]^

#### 2.1.2 Micro‐ and Nano‐Bots Driven by Optical Actuation

Optically driven MMBs and MNBs convert light into motions that can be controlled by wavelength, irradiation angle, and intensity of the light source, as well as the optical responsiveness of the materials utilized in the bots’ structures.^[^ ^40^, ^52^, ^73^ ^]^ Semiconducting materials, such as titanium dioxide (TiO_2_), zinc oxide (ZnO), and cadmium sulfide (CdS),^[^ ^47^ ^]^ as well as liquid crystal polymers, including azobenzene‐,^[^ ^74^ ^]^ diarylethenes‐,^[^ ^75^ ^]^ and spiropyrans‐containing polymers,^[^ ^76^ ^]^ are among the most applicable materials for designing optical actuation systems. The actuation mechanisms of these materials are based on photo‐generation of electron/hole pairs^[^ ^77^ ^]^ or configurational shape transition upon light absorption (e.g., the trans–cis photoisomerization of azobenzene‐based polymers).^[^ ^78^ ^]^ The latter could exhibit a response to various wavelengths, ranging from UV to visible light, depending on bandgaps and electronic structures of materials.^[^ ^52^ ^]^ The fabrication method of choice in this case is the two‐photon polymerization technique, which relies on the absorption of two photons triggered by a focused laser pulse and applies to a photosensitive material, enabling an ultra‐high resolution and precise fabrication method for 3D complex structures.^[^ ^79^, ^80^ ^]^ Light‐responsive bots triggered by visible or UV rays to move by changing shape are not the only option. Photocatalytic‐ and photothermal‐based bots employ photo‐triggered chemical reactions and localized heat generation to generate chemical gradients or localized temperature gradients, which in turn drive motion.^[^ ^75^ ^]^ Advantages of the optical MMBs and MNBs include high temporal and spatial resolution, ease of operation, scalable fabrication, and capability for real‐time optical tracking. However, the lower surface penetration capability of the optical field could hinder broader application in vivo. These bots are nevertheless highly advantageous in research lab settings in combination with organelles or organ‐on‐a‐chip platforms with a small depth required penetration depth and semi‐transparent environment.^[^ ^40^, ^81^ ^]^

#### 2.1.3 Micro‐ and Nano‐Bots Driven by Acoustic Actuation

Acoustically driven MMBs and MNBs, also known as ultrasonically propelled bots, offer access to otherwise hard‐to‐reach sites of the body, outstanding biocompatibility, deep tissue penetration, and real‐time tracking.^[^ ^48^, ^82^ ^]^ An acoustic field is applied to MMBs and MNBs, which have asymmetric structures, to produce unbalanced pressure forces and generate net momentum that pushes the bot forward.^[^ ^83^, ^84^ ^]^ Sharp‐edge‐like tails, which can swing with a helical and planar wave resonance and drive rotational and linear motions, are another popular geometry.^[^ ^83^ ^]^ Despite the potential of acoustically driven bots, this field is still in its early stages, with relatively few studies published to date. Significant challenges remain, particularly in developing advanced materials suited for acoustic propulsion and refining acoustic guidance systems capable of precisely controlling direction in complex environments.^[^ ^83^, ^85^, ^86^ ^]^

#### 2.1.4 Micro‐ and Nano‐Bots Driven by Electrical Actuation

Electrically actuated miniature robots offer precise positional control, tunable speed, high responsiveness, and integration ability with electronics.^[^ ^50^, ^52^ ^]^ Notable challenges are the requirement for high voltage in some cases, the high electrical conductivity of biological media that can interfere with the bot's functions, and sensitivity to environmental factors such as pH, temperature, and fluid flow, all of which can negatively affect their performance.^[^ ^87^, ^88^ ^]^ Electrically actuated mini‐robots are either electric field‐actuated or electric current‐actuated.^[^ ^87^ ^]^ Electric field‐driven bots consist of materials with an intrinsic charge, responding to the external electrical field generated by uniform direct current (DC) or alternating current (AC).^[^ ^89^ ^]^ This class of electrical robots typically includes metallo‐dielectric Janus particles, such as gold (Au)‐polystyrene microspheres or cadmium‐polypyrrole nanowires, which move via electroosmotic flow, electrophoresis, or electrolyte diffusiophoresis mechanisms.^[^ ^90^, ^91^, ^92^ ^]^ The speed and direction of these bots can be controlled with high resolution by adjusting their zeta potential as well as the strength and frequency of the external electric field.^[^ ^90^, ^93^, ^94^ ^]^

Advances in nanofabrication techniques and piezoelectric materials have enabled current‐actuated MMBs and MNBs.^[^ ^95^ ^]^ When a voltage is applied, these robots demonstrate an inverse piezoelectric effect, which converts the input electrical energy to agile linear and rotational mechanical movements.^[^ ^96^ ^]^ The main advantage of current‐actuated MMBs and MNBs is their ability to exhibit self‐propulsion, which can result in the development of intelligent miniature robots and drive future advances in the field.^[^ ^97^ ^]^

### 2.2 Chemical Propulsion

Chemically driven MMBs and MNBs rely on generating energy by chemical reactions and translating it to mechanical movements. These are simple, cost‐effective, and do not rely on complex external drive mechanisms.^[^ ^98^, ^99^ ^]^ These types of robots can be classified based on their actuation mechanisms into three main categories, namely, self‐electrophoretic,^[^ ^100^ ^]^ self‐diffusiophoretic,^[^ ^101^ ^]^ and bubble propulsion motions.^[^ ^102^, ^103^ ^]^

Self‐electrophoretic MMBs represent the earliest subgroup of chemically powered bots, relying on local electric fields generated by redox reactions and chemical gradients for autonomous propulsion.^[^ ^87^, ^104^ ^]^ Examples include bimetallic nanowires such as Au‐platinum (Pt) or Au‐Nickle (Ni) nanorods that undergo decomposition reactions in the presence of hydrogen peroxide and produce ions asymmetrically, which ultimately results in forward motion.^[^ ^105^ ^]^

Self‐diffusiophoretic bots are commonly used in combination with Janus architectures, which leads to motion through establishing a heterogeneous concentration gradient.^[^ ^101^, ^102^ ^]^ In these bots, asymmetric chemical composition or morphology generates fluid flows from low to high solute concentration regions, creating a pressure distribution that propels the bot in the opposite direction.^[^ ^106^ ^]^ The outstanding feature of these types of bots compared to self‐electrophoretic bots is their ability to operate in high ionic strength environments. However, the propulsion force in self‐diffusiophoretic bots is typically weaker, resulting in slower movement.^[^ ^102^ ^]^

To overcome these limitations, bubble‐propulsion MMBs and MNBs have been developed.^[^ ^107^ ^]^ These bots generate microstreaming of bubbles through catalytic reactions with their fuel source, such as hydrogen peroxide (H_2_O_2_) decomposition, metal oxidation (e.g., magnesium (Mg), aluminum (Al), and iron (Fe)), or water (H_2_O) electrolysis.^[^ ^107^, ^108^ ^]^ An important aspect of their design is that by reducing the bots' size, the surface tension and Laplacian pressure of generated bubbles will increase, which in turn can lead to dominating the inertia force of fluid around the bot and the bots' movement.^[^ ^109^, ^110^ ^]^

### 2.3 Biological Propulsion

Biologically driven bots, also known as biohybrid robots, utilize living organisms as their actuators. Advantages include self‐repair, self‐replication, real‐time stimuli response, flexibility, high power density, durability, and self‐powered motions.^[^ ^111^, ^112^ ^]^ Biohybrid MMBs and MNBs are mainly composed of biomotors that are integrated with artificial constructs and designed specifically for applications such as cargo delivery or enhanced spatial maneuverability in medical settings. These synthetic components can be attached to their living actuators via covalent^[^ ^113^ ^]^ or non‐covalent interactions,^[^ ^114^ ^]^ physical entrapment,^[^ ^115^ ^]^ and internalization.^[^ ^116^ ^]^ As illustrated in Figure 1, biohybrid MMBs are classified based on their natural biomotor, namely spermbots,^[^ ^117^, ^118^ ^]^ bacteriobots,^[^ ^119^ ^]^ algae‐based bots,^[^ ^35^ ^]^ and macrophage‐based bots.^[^ ^116^ ^]^

Spermbots, which are designed based on the flagellar movement of spermatozoa (sperm cells), are promising for gynecological healthcare applications.^[^ ^117^, ^120^ ^]^ Spermbots utilize sperms as motile cells to swim via their snake‐like flagellar motion in different patterns (typical, chiral ribbons, or helical), which can be controlled by their own natural function or through external stimuli. Spermbots demonstrate thermotaxis (ability to move toward a temperature gradient), chemotaxis (ability to move toward a higher concentration of extracellular signals), and rheotaxis (ability to move against fluid flow), thereby enabling them to move quickly and naturally in a controlled manner.^[^ ^121^ ^]^ Various research groups have explored linking single sperm cells to various types of stimuli‐responsive agents, for instance, magnetic microtubes^[^ ^114^, ^115^, ^122^ ^]^ or pH‐responsive microstructures,^[^ ^123^ ^]^ to enhance their targeting capabilities; however, low coupling efficiency remains a challenge.^[^ ^117^ ^]^

Bacteria‐based bots, called bacteriobots, are another type of biohybrid MMBs that utilize the flagellar propulsion of various highly motile microorganisms, such as Salmonella, Streptococcus, Escherichia, Proteus, Caulobacter, Clostridium, and Listeria, for fast and versatile motion.^[^ ^124^, ^125^ ^]^ To enhance the cargo‐loading capacity and penetration depth of these bacteriobots, they are generally merged with components such as polymeric tubes, liposomes, or nanoparticles, by employing various bioconjugation techniques.^[^ ^113^, ^124^ ^]^ For example, some bacteria strains accumulate in the hypoxic microenvironment of solid tumors, making them applicable for drug delivery to tumors.^[^ ^119^, ^126^, ^127^ ^]^ Bacteriobots face a variety of challenges, including toxicity, low efficiency due to immune system clearance, and complex culturing processes. However, synthetic biology may offer some practical solutions that overcome these issues.^[^ ^128^, ^129^ ^]^

Another class of attractive biohybrid MMBs is algae‐based robots. These bots move based on multiple mechanisms, including photosynthesis and flagellum beating, and can be applied for bioimaging, drug delivery, and photodynamic therapy.^[^ ^130^, ^131^ ^]^ For creating algae‐based MMBs, various types of algae cells, including green microalgae (e.g., *Chlamydomonas reinhardtii*),^[^ ^132^ ^]^ diatoms (e.g., *Thalassiosira weissflogii*),^[^ ^133^ ^]^ and dinoflagellates (e.g., *Pyrocystis lunula* ^[^ ^134^ ^]^) can be employed. Attractive characteristics of algae‐based MMBs include the rich chemistry of their cell walls that offers a range of bioconjugation options, fast movement, scalability due to rapid growth, and no inherent toxicity. Employing them for practical applications, however, could be challenging due to their complicated controllability, especially in deep tissue that light cannot penetrate, and their sensitivity to environmental conditions, particularly pH.^[^ ^130^, ^135^, ^136^ ^]^

Macrophage‐based MMBs initially garnered attention for targeted cancer therapy but also exhibit characteristics attractive for infection management.^[^ ^116^, ^137^ ^]^ Macrophages offer excellent cargo‐loading capabilities, high throughput and effective delivery due to their intrinsic engulfing function (aka phagocytosis), high compatibility with the immune system, and efficient penetration of various biological barriers.^[^ ^137^, ^138^, ^139^ ^]^ As a result, macrophage‐based MMBs are promising for the treatment and diagnosis of infectious diseases, although no report on this topic exists to date.^[^ ^140^, ^141^ ^]^

## 3 Application of Micro‐ and Nano‐Bots for Efficient Infection Management

**Figure 2:**
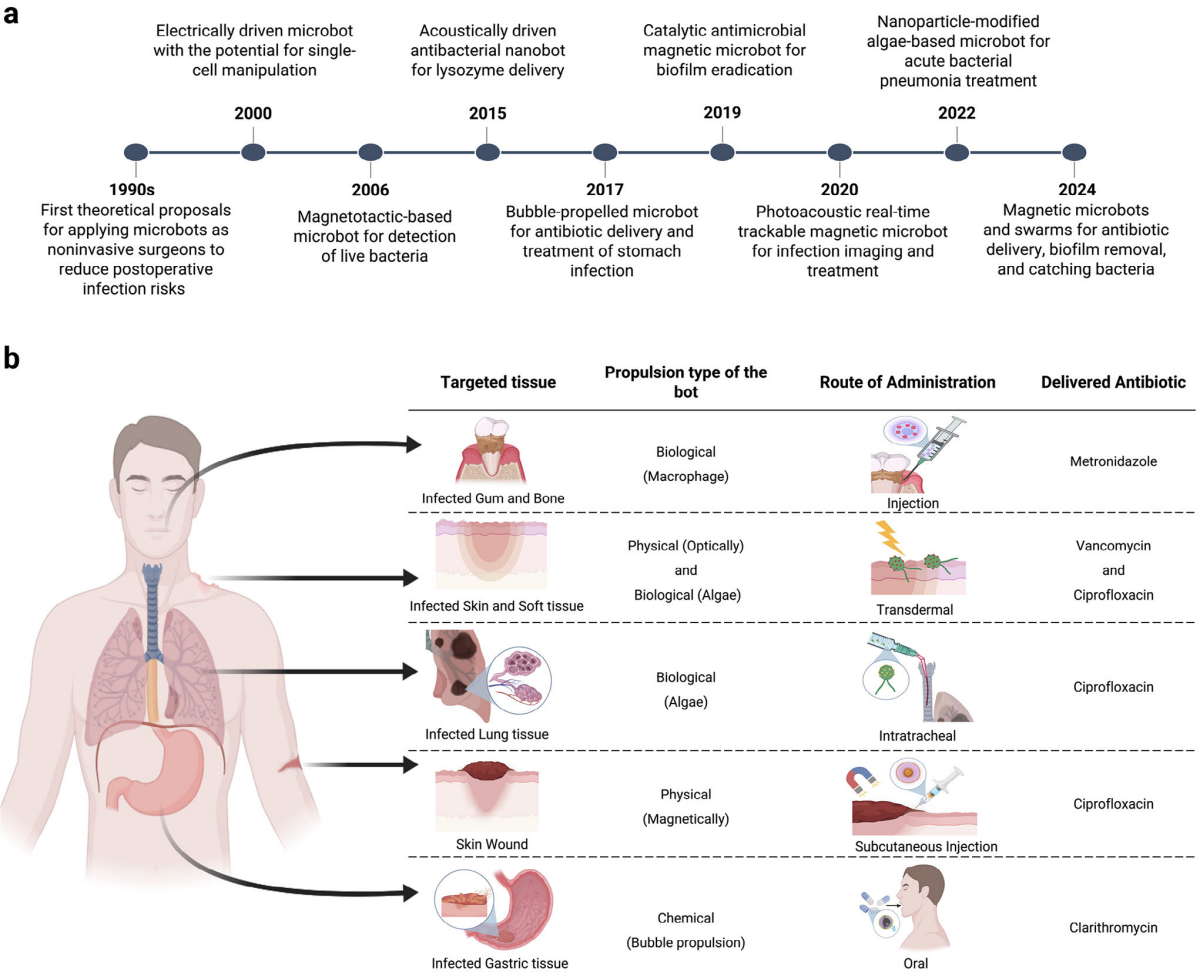
a) Overview of the progress made in the development of MMBs and MNBs for infection management, reported with respect to chronological milestones. References: First theoretical studies developing initial MMBs^[^ ^144^, ^233^ ^]^; MMBs with potential for single‐cell manipulation^[^ ^23^ ^]^; First magnetotactic‐based MMB for bacteria detection^[^ ^213^ ^]^; Acoustically driven MNB for lysozyme delivery^[^ ^179^ ^]^; Bubble‐propulsive MMB for antibiotic delivery^[^ ^166^ ^]^; Antibiofilm magnetic MMB^[^ ^183^ ^]^; Photoacoustic Imaging‐Trackable MMB^[^ ^21^ ^]^; Algae‐based MMB for lung infection^[^ ^35^ ^]^; Multimodal magnetic MMB for antibiotic delivery and biofilm eradication^[^ ^186^ ^]^ and magnetic micro‐swarms for capturing bacteria.^[^ ^234^ ^]^ b) Utilizing MMBs and MNBs as novel carriers for traditional antibiotics. MMBs and MNBs can be administrated via various routes, including transdermal,^[^ ^20^ ^]^ oral,^[^ ^166^ ^]^ intratracheal,^[^ ^35^ ^]^ and injection.^[^ ^165^, ^167^ ^]^ Currently, different research groups have successfully delivered Metronidazole, Ciprofloxacin, and clarithromycin by applying biological, physical, and chemical‐based MMBs, respectively.^[^ ^165^, ^166^, ^167^ ^]^

As demonstrated in **Figure** **2**a, the conceptual development of MMBs and MNBs that started in the 1990s. Following the technological revolution enabled by the advancements in micro and nanotechnology, these theoretical investigations led to numerous remarkable advancements in designing MMBs and MNBs. Here, we outline the application of MMBs and MNBs at different stages of infection occurrence, including prevention (e.g., reducing the risk of surgical site infection), early and precise diagnosis (e.g., employing MMBs and MNBs as efficient biosensors and bio‐imaging agents), and treatment (e.g., by using MMBs and MNBs as effective targeted delivery systems or anti‐biofilm machines).

### 3.1 Minimally Invasive Surgery for Infection Prevention

Surgical site infection is a significant challenge that is attributed to bacteria inoculation into the incision site during the time of surgery when both the main biological barriers and the immune system are compromised.^[^ ^142^ ^]^ Applying MMBs and MNBs as minimally invasive surgeons is a revolutionary strategy for preventing surgical infection.^[^ ^143^, ^144^ ^]^ Srivastav et al. designed magnetic MMBs composed of calcified porous biotubes coated with an iron‐titanium layer, which precisely targeted a single cell, drilled into them, and performed microsurgery at the single‐cell level. The results of this study not only revealed the promising effects of MMBs for the reduction of surgical infection risks but also offered a novel approach for removing infected cells via these microsurgeons.^[^ ^145^ ^]^ Others have demonstrated the outstanding potential of magnetic MMBs for conducting minimally invasive surgeries at the posterior segment of the eye and the cochlea of the inner ear for the treatment of ophthalmic diseases and hearing loss, respectively.^[^ ^146^, ^147^ ^]^ Developing MMBs and MNBs as the next generation of surgeons reduces risks associated with surgical incisions and post‐operative pain, performing precise and on‐demand surgery procedures on a microscale and decreasing post‐surgical infections.^[^ ^145^, ^146^, ^147^, ^148^ ^]^

### 3.2 Efficacious Tools for Imaging Infection

Effective imaging enables real‐time monitoring of the efficiency of the infection treatment strategies, helps avoid excessive antibiotic prescription, and ultimately aids accurate and targeted treatment for infectious diseases.^[^ ^149^ ^]^ The conventional anatomical modalities that are utilized for imaging infected tissues are computed tomography (CT), photoacoustic imaging (PAI), magnetic resonance imaging (MRI), positron emission tomography (PET), and fluorescence imaging (FLI). These all face limitations, such as inadequate tissue penetration and low spatial resolution.^[^ ^150^, ^151^ ^]^ MMBs and MNBs promise to improve these imaging techniques by serving as targeted contrast agents’ carriers that precisely distinguish bacterial infection from other sterilized inflammation sites.^[^ ^151^ ^]^ This is especially advantageous in deep tissues made accessible by their small size and active propulsion.^[^ ^21^, ^152^, ^153^, ^154^ ^]^

As an example, to conduct a high‐resolution and real‐time visualization of the intestine while applying a treatment agent, Wu et al. designed chemical MMBs, working based on the Mg reactions and bubble propulsion mechanism, that could effectively navigate by using a PAI technique. The results of this study demonstrated that employing these chemical MMBs as cargo delivery systems allowed for precise navigation of the drug delivery process, enhancement of the PAI contrast with the deposited Au layer on these MMBs, and increased retention times in the harsh gastric environment.^[^ ^153^ ^]^ In another study, magnetic MMBs loaded with iodinated X‐ray contrast agents and therapeutic agents were guided via magnetic actuation under real‐time X‐ray imaging. These MMBs exhibited outstanding potential for tracking by MRI as a postoperative imaging technique, which enabled monitoring the safety and accuracy of the intervention.^[^ ^155^ ^]^ MMBs and MNBs have been used for multiple simultaneous functions, namely as imaging tools and drug delivery carriers. Regarding this, Xie et al. designed magnetic‐biohybrid MMBs, composing Spirulina algae coated with magnetite nanoparticles (Fe_3_O_4_ NPs) and polydopamine, and applied them for real‐time image tracking by PAI and photothermal therapy against pathogenic bacterial infection such as Klebsiella pneumoniae.^[^ ^21^ ^]^ The polydopamine coating resulted in strong near‐infrared absorption of these MMBs that allowed for effectively navigation as well as precise treatment resulting from generating focused heat.^[^ ^21^ ^]^ Finally, it should be mentioned that regardless of the fascinating potential of MMBs and MNBs for facilitating the imaging process of infected regions, not many studies have been published on this subject.

### 3.3 Sensitive Biosensors for Diagnostics

Developing highly sensitive and rapid biosensors for the early detection of infectious agents is crucial strategy for controlling infectious diseases.^[^ ^10^ ^]^ Having controlled locomotion and active mass transfer capabilities, MMBs and MNBs are poised as the next generation of ultra‐sensitive biosensors with offering several advantages, namely multifunctionality, fast response and enhanced detection. Multifunctionality enables sensing and capture of infectious agents while simultaneously delivering drugs. Rapid movement allows for a faster response rate and reduced bioassay time, and engineered surface chemistry enables detection a wider range of infectious agents.^[^ ^156^, ^157^, ^158^ ^]^

Exhibiting active locomotion, bots can sense analytes via mechanisms that depend on motion.^[^ ^156^, ^159^ ^]^ To detect the *Clostridiodes difficile* (C.diff) infection in the gastrointestinal tract, Zhang et al. designed highly sensitive biosensors consisting of magnetic MMBs conjugated with fluorescent probes, which could effectively detect toxins secreted by C. diff bacteria via the motion speed and rate of the fluorescence quenching.^[^ ^160^ ^]^ In another study, chemical MNBs, composed of platinum antibody‐coated nanoparticles, were applied for the diagnosis of the Zika virus based on the same mechanism. Accumulation of platinum MNBs on the surface of Zika viruses resulted in the movement of these aggregates, and the concentration of the virus was calculated based on the changes in bots' motion.^[^ ^161^ ^]^

MMBs and MNBs can significantly improve the efficiency of conventional biosensors. By stirring samples and enhancing mass transfer, they can promote faster and more efficient interactions with infectious agents.^[^ ^162^, ^163^, ^164^ ^]^ In an electrochemical biosensor for the detection of SARS‐CoV‐2 virus, magnetic MMBs conjugated with SARS‐CoV Spike antibody enhanced the micro‐mixing effect in the test tubes, which in turn increased the selectivity of the biosensor and reduced the response time compared to conventional ELISA immunoassays.^[^ ^162^ ^]^ **Table** **2** outlines the structures of various MMBs and MNBs used for the detection of infectious agents and their transduction mechanism.

**Table 2:** The structures and method of transduction of some MMBs and MNBs applicable for infection diagnosis.

Type of the robot	Mechanism of propulsion	Structure of the robot	Detected infectious agent	Limit of Detection (LOD)	Method of transduction	Refs.
MMB	Physical‐ Magnetic	*G. lucidum* spores that were first coated by Fe_3_O_4_ nanoparticles and then conjugated with fluorescent carbon dots	Bacteria ‐ *C. diff*	1.73 ng mL^−1^	Fluorescent biosensing that simultaneously occurred with motion‐based sensing	[160]
MMB	Physical‐ Magnetic	Dynabeads^®^M‐280 (polystyrene beads coated with a polyurethane layer) and Au‐Ag (core–shell) nanorods that were coated with SARS‐CoV‐2019 Spike antibody were applied together for detection via an immuno‐sandwich complex	Virus – COVID19	1.11 PFU mL^−1^	Electrochemical biosensing	[162]
MMB	Biological and Physical‐ Bacteriobot and Magnetic	Fe_3_O_4_ and CRISPR/Cas12a system were immobilized on genetically modified green fluorescent labeled *E. coli* bacteria	Viruses – Decapod iridescent and White spot syndrome viruses	7 copies µL^−1^	Fluorescent biosensing	[228]
MMB	Biological‐Bacteriobot	MO‐1 magnetotactic bacteria were decorated with rabbit anti‐MO‐1 cell polyclonal antibodies	Bacteria – *S.aureus*	–	Visualization via phase contrast microscopy	[212]
MNB	Chemical‐ Self‐electrophoretic	Platinum nanoparticles were conjugated with monoclonal anti‐Zika virus antibody	Virus‐ Zika Virus	1 particles µL^−1^	Motion‐based sensing	[161]
MNB	Physical‐ Magnetic	Fe_3_O_4_ nanoparticles, which were first coated by Au seeds and Ag particles and then covered with DNA probes	Virus – COVID19	6.1 ng mL^−1^	Electrochemical biosensing	[163]
MNB	Physical‐ Magnetic	Luminescent reporter T4 bacteriophages were covalently conjugated with azide‐decorated magnetic nanoparticles	Bacteria ‐ *E. coli*	<10 CFU/100 mL	Luminescent biosensing	[229]
MNB	Physical‐ Magnetic	Iron oxide/silica (core–shell) nanoparticles were conjugated with cell wall‐binding domain (CBD)‐endolysin sensors	Bacteria – *S.aureus*	10^5^ cell mL^−1^	Fluorescent biosensing	[164]

### 3.4 Targeted Delivery Systems for Antimicrobials

The ability to precisely control spatial and temporal control over the movements of MMBs and MNBs makes them ideal platforms for carrying antimicrobial compounds, including antibiotic drugs or anti‐infective biologics. As demonstrated in Figure 2b, MMBs and MNBs exhibited great potential for loading various antibiotics and efficiently delivering them to a wide range of infected tissue including gums, bones, skin, and so forth.^[^ ^20^, ^35^, ^165^, ^166^, ^167^ ^]^ For instance, different studies revealed the outstanding potential of magnetically and algae‐based MMBs for delivering ciprofloxacin, one of the frequently used antibiotics, to treat the infection of skin, soft tissues, and lungs, respectively.^[^ ^20^, ^35^, ^167^ ^]^ Bubble propulsion‐based MMBs have been reported for loading clarithromycin and treating gastrointestinal tract infections through oral administration.^[^ ^166^ ^]^ Ability to deliver antimicrobials directly to infected target cells mitigates the loss of antibiotics that occurs through systemic administration. In addition, bots’ ability to penetrate through various biological barriers reduces the required time for reaching to infected tissues, all of which can significantly improve the efficacy of traditional antibiotics.^[^ ^165^, ^166^, ^167^, ^168^ ^]^

**Figure 3:**
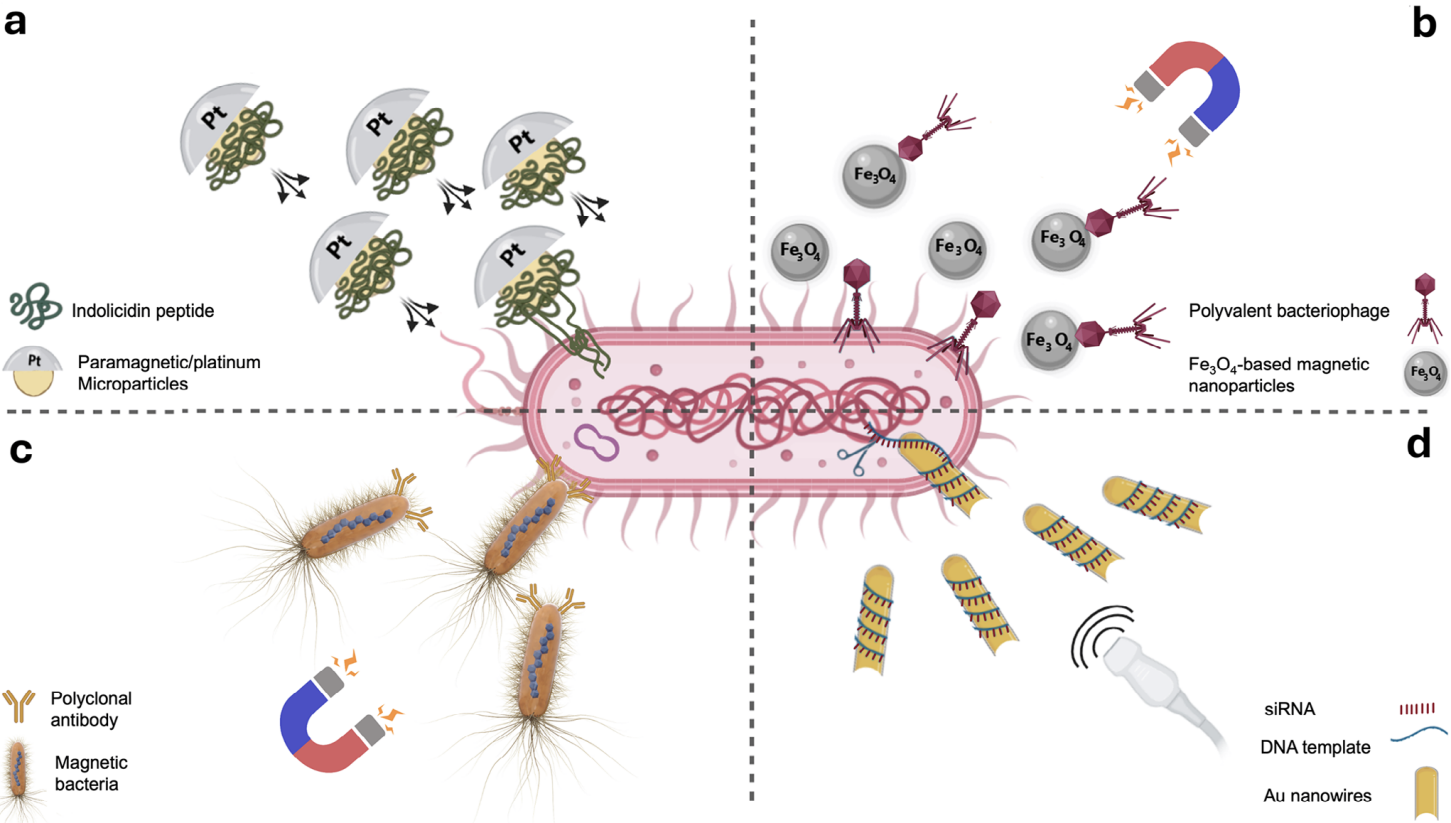
Applying MMBs and MNBs as targeted delivery systems for anti‐infective biological agents. a) Self‐diffusiophoretic chemical MMBs as the carrier of anti‐bacterial peptide;^[^ ^169^ ^]^ b) Magnetic MNBs for bacteriophage delivery;^[^ ^173^ ^]^ c) Antibody‐coated magnetotactic bacteria for targeting bacteria cells;^[^ ^170^ ^]^ d) Acoustically MNBs for delivery of siRNA.^[^ ^178^ ^]^

The outstanding potential of MMBs and MNBs as targeted delivery systems is not only limited to the delivery of antibiotics but can also be useful for anti‐infective biological agents. The prevalence of multidrug‐resistant pathogens necessitates the development of novel anti‐infective agents, including therapeutic peptides,^[^ ^169^ ^]^ antibodies,^[^ ^170^ ^]^ DNA‐ and RNA‐vaccines,^[^ ^171^ ^]^ CRISPR Cas9 systems,^[^ ^172^ ^]^ bacteriophages.^[^ ^173^, ^174^, ^175^, ^176^ ^]^ Biological barriers and the host immune system hamper effective delivery of high does these biologics to the site of infection. This is where MMBs and MNBs can help.^[^ ^169^, ^171^, ^173^, ^177^ ^]^ As shown in **Figure** **3**, recent studies reported the application of MMBs and MNBs as targeted carrier systems for delivering therapeutic peptides,^[^ ^169^ ^]^ bacteriophages,^[^ ^173^ ^]^ antibodies,^[^ ^170^ ^]^ and small interfering RNA (siRNA).^[^ ^178^ ^]^ Self‐diffusiophoretic chemical MMBs, composed of paramagnetic/platinum Janus particles coated with indolicidin peptide, were utilized for the treatment of Staphylococcus aureus infection. Applying MMBs not only enabled targeted delivery but also increased the dose of peptides delivered into bacterial biofilms through mechanical action and prolonged contact of peptide‐coated MMBs with bacteria (Figure 3a).^[^ ^169^ ^]^ In another report, acoustically‐propelled MNBs composed of porous gold nanowires was used for the delivery of lysozyme, an antibacterial glycoside‐hydrolase enzyme, which led to the inactivation bacteria within a few minutes.^[^ ^179^ ^]^ Bacteriophages (bacterial viruses) can also be delivered by utilizing MMBs and MNBs.^[^ ^180^, ^181^ ^]^ The results of a study that conjugate polyvalent bacteriophage with chitosan‐coated Fe_3_O_4_ nanoparticles demonstrated a higher phage concentration in targeted sites was achievable with magnetic control of nanoparticle movement (Figure 3b).^[^ ^173^ ^]^ The immense promise of biologics, however, is hindered by challenges with their stability through processes inherent to the manufacturing of bots and must be overcome as a prerequisite for effective implementation.^[^ ^6^, ^174^, ^176^, ^182^ ^]^

MMBs and MNBs can also be coated with anti‐infective agents and biorecognition molecules such as antibodies to improve their targeting ability. As illustrated in Figure 3c, magnetotactic bacteria coated with anti‐MO‐1 polyclonal antibodies were shown to accurately target *S. aureus* by generating a mechanical force under a swing magnetic field.^[^ ^170^ ^]^ Another report applied gold nanowires as acoustically actuated MNBs to deliver siRNA in a controlled fashion and increase knockdown efficiency of the targeted cells. Although this project was conducted on eukaryotic cells, it still reveals the potential of MNBs for DNA or RNA delivery and intracellular gene delivery that can be utilized for the treatment of various infectious diseases (Figure 3d).^[^ ^178^ ^]^

### 3.5 Biofilm Eradication Micromachines

**Figure 4:**
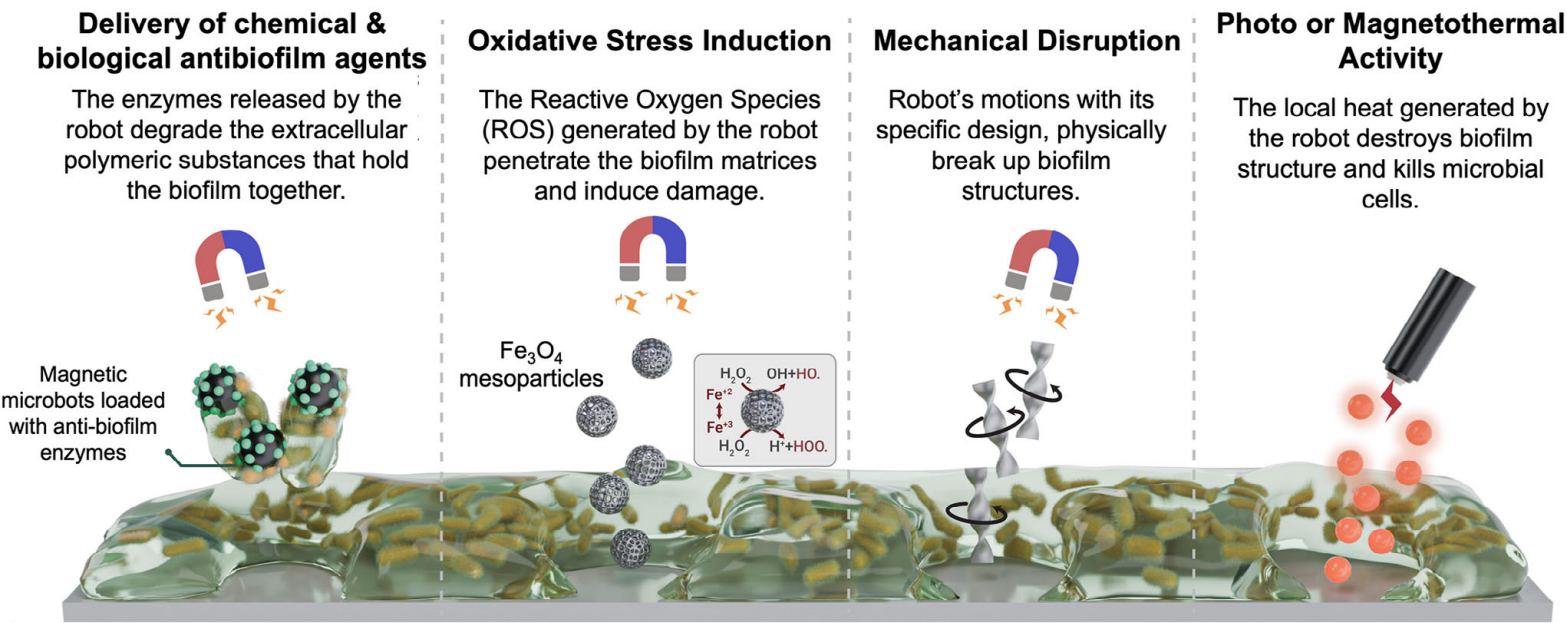
Different mechanisms of MMBs and MNBs for eradicating biofilm integration, including delivery of anti‐biofilm agents,^[^ ^184^ ^]^ inducing oxidative stress by creating reactive oxygen species,^[^ ^183^ ^]^ disruption of biofilm by mechanical forces,^[^ ^186^ ^]^ and generating local heat based on photothermal activity.^[^ ^185^ ^]^

Utilizing MMBs and MNBs as biofilm eradication machines is another significant aspect of their role in infection treatment. As shown in **Figure** **4**, MMBs and MNBs can eradicate biofilm through multiple mechanisms, namely delivery of antibiofilm agents, mechanical disruption of the biofilm structure, oxidative stress induction, and photo or magneto thermal activity.^[^ ^108^, ^167^, ^183^, ^184^, ^185^ ^]^ Magnetic MMBs, composed of halloysite nanotubes and Fe_3_O_4_ nanoparticles, were reported to provide switchable spinning and vortex motions induced by a magnetic field. These bots generated a considerable mechanical force that disrupted the extracellular matrix of S. aureus biofilms on titanium mesh of bone restoration implants.^[^ ^186^ ^]^ Bubble‐propulsion chemically actuated MMBs, comprised of manganese oxide nanosheets, have also been reported to be effective at rupturing P. aeruginosa biofilms by generating O_2_ bubbles.^[^ ^108^ ^]^

Induction of oxidative stress can generally lead to the formation of bacterial biofilms, but if induced under the right conditions, it can cause biofilm eradication. MMBs, composed of Fe_3_O_4_ nanoparticles have been reported to catalyze hydrogen peroxide (H_2_O_2_) and generate reactive oxygen species (ROS) that degrade the exopolysaccharide of the biofilm structure.^[^ ^183^ ^]^ Magnetic MMBs, composed of ferric oxide (Fe_2_O_3_) helical microparticles, have also been developed with peroxidase‐mimicking performance under magnetic actuation, generating local ROS and degrading the biofilm formed in tympanostomy tubes.^[^ ^187^ ^]^

MMBs and MNBs have been developed that exploit multiple mechanisms for eradicating biofilms. Optically actuated MMBs, composed of TiO_2_ nanotubes and CdS nanoparticles immobilized with urease enzyme, have been reported to remove 90% of bacterial biofilm through multiple mechanisms, including urease delivery to hydrolyze the biofilm, ROS production, and mechanical disruption through movement.^[^ ^184^ ^]^ In addition, magnetothermal and photothermal effects that induce local heat generation can further increase the biofilm permeability for bots and the effectiveness of other anti‐infective agents.^[^ ^185^, ^188^ ^]^ Optical MNBs, consisting of mesoporous‐silica nanoparticles coated with a gold layer, were reported to generate considerable photothermal activity after exposure to near‐infrared light irradiation. This photothermal effect led to the development of an asymmetric temperature gradient that led to biofilm eradication.^[^ ^185^ ^]^

## 4 Challenges for Employing Micro‐ and Nano‐Bots for Infection Management and Mitigating Strategies

MMBs and MNBs have outstanding potential to be used in infection prevention, diagnostics, and treatment, however, they face some limitations in terms of triggering an immune response and moving in hard‐to‐reach niches, including deep infected tissues. In this section, we discuss potential obstacles to designing MMBs and MNBs and propose strategies to address them.

### 4.1 Minimizing Immune Response to Overcome Immune Barriers

**Figure 5:**
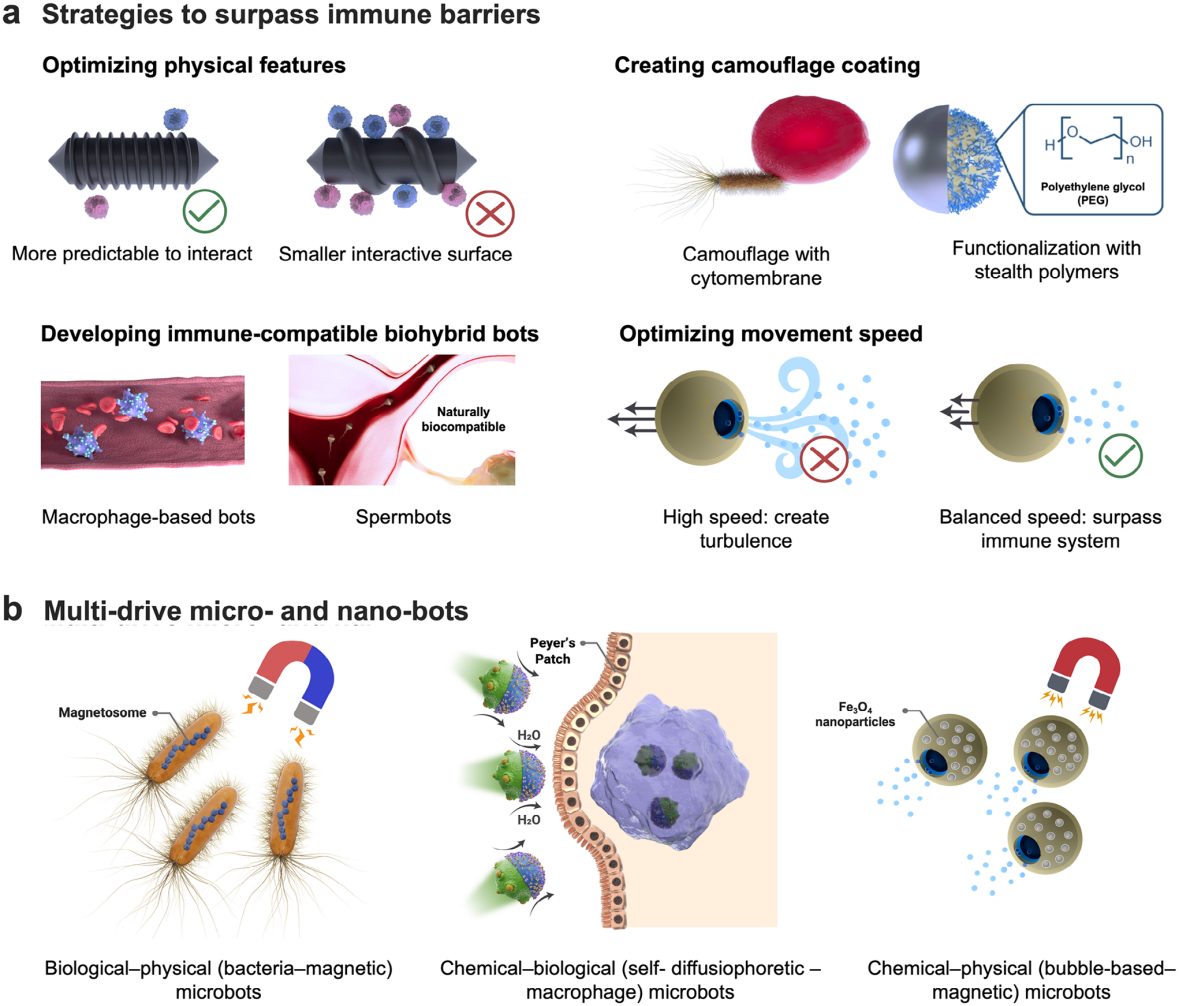
a) Four strategies by which the immunomodulation of MMBs and MNBs improves are optimization of the physical features and movement speed of MMBs and MNBs, creating camouflage coating by stealth polymers or cell membranes, and developing immune compatible biohybrid bots such as spermbots or macrophage‐based bots. b) Examples of multi‐drive MMBs and MNBs that can be classified based on the propulsion mechanisms they combined. In this regard, they can be categorized as biological–physical, biological–chemical, and chemical–physical bots. As an example of biological–physical MMBs, magnetotactic bacteria^[^ ^213^ ^]^ are depicted, which enjoy their natural flagella movements and can be guided by the magnetic field due to the presence of magnetosomes in their structures. To exemplify chemical–biological MMBs, twin‐engine self‐diffusiophoretic – macrophage‐based MMBs^[^ ^139^ ^]^ are shown. These bots are prepared from Janus yeast, whose half of its surface is covered with enzymes, and enjoy in situ switching to the macrophage engine in Peyer's patch. Finally, the bubble‐based – magnetic MMBs^[^ ^214^, ^231^ ^]^ are demonstrated as an illustration of chemical–physical bots.

Engineering materials for modulating desirable immune responses is one of the essential steps in designing novel biomaterials, and MMBs and MNBs are no exception. The strategies that can be employed for tailoring the immune response of MMBs and MNBs are discussed in this section along with select examples (**Figure** **5**a).

Camouflage coating on the bots' surfaces is the most common approach for immune evasion.^[^ ^189^ ^]^ Several studies report on functionalizing MMBs and MNBs with stealth polymers, especially polyethylene glycol (PEG) chains, for reducing the bots' immunoreactivity.^[^ ^190^, ^191^, ^192^ ^]^ Incorporating PEG moieties on the bots' surface resulted in the creation of a strong hydrophilic layer that inhibited protein absorption and immune activation.^[^ ^189^, ^190^ ^]^ In addition to coating with stealth polymers, modifying the surface of MMBs and MNBs with naturally biocompatible cell membrane components is another approach to evade the immune system.^[^ ^193^, ^194^ ^]^ For instance, the results of a study that observed the immune‐evasive properties of bacteriobots showed that attaching erythrocytes to these MMBs can effectively aid immune evasion and increase their circulation time.^[^ ^193^ ^]^

Another strategy for modulating the immune response of MMBs and MNBs is tailoring their physical features.^[^ ^189^, ^195^ ^]^ For instance, the results of a study that focused on optimizing structural characteristics of helical magnetic MMBs for reducing their immunoreactivity demonstrated that increasing the helix turn numbers decreased immune response. This can be explained by the fact that physical interaction and chemical interactions with macrophages are dependent on the morphology and surface area of MMBs.^[^ ^195^ ^]^ The morphology of the MMBs and MNBs can significantly affect their speed, which is itself known to affect immune response. Therefore, investigating the combined effects of movement speed and morphology appears to be an important area of investigation to unlock future advancements in this field.

Employing immune‐compatible biohybrid MMBs composed of immune‐compatible cells, including sperm and macrophage, is another promising strategy.^[^ ^196^ ^]^ Multiple reports have demonstrated the outstanding immune compatibility of spermbots for application in the uterus and ovary tissues, owing to their inherent swimming ability in this niche.^[^ ^121^, ^197^ ^]^

Optimizing movement speed can tailor immune responses to bots. It should be noted that high‐speed bots can generate chaotic flows and turbulent regions,^[^ ^198^ ^]^ which can result in the localized activation of immune cells.^[^ ^199^ ^]^ Therefore, speed is a factor that must be optimized, and higher speed is not necessarily desirable in all application scenarios. It is also worth noting that regardless of the importance of this issue, which is the specific feature of medical bots compared to other biomaterials, the number of studies conducted on this subject is very limited, so further research is needed.

### 4.2 Navigating Micro‐ and Nano‐Bots in Deep Tissue

One of the main challenges in infection management is treating and imaging deep tissue infections, which requires the patient to undergo invasive treatment methods, including drainage and debridement surgery.^[^ ^200^ ^]^ If designed properly, MMBs and MNBs can offer a non‐invasive path for treating deep‐tissue infections. Research points to the driving force as one of the important parameters to optimize for increasing the penetration depth of MMBs and MNBs. The penetration depth of physical bots is believed to be strongly dependent on the power and transparency of the navigator field. Magnetic and acoustic MMBs and MNBs have the potential to navigate deeper into tissue compared to their optical counterparts.^[^ ^40^, ^201^ ^]^ The penetration depth of chemical MMBs and MNBs can be estimated based on their lifetime, which depends on their chemical composition/fuel source and geometry. For instance, the results of a study conducted by Zhou et al. on bubble propulsion chemical MMBs revealed that changing the chemical composition of these MMBs from Au‐Zn to Fe‐Zn, thus reducing the difference in electrochemical potential difference, increased the bot's lifetime. Altering the physical structure of the same MMB and applying a solid structure instead of a hollow one also increased the lifetime.^[^ ^202^ ^]^ The penetration depth of biohybrid MMBs and MNBs is completely dependent on the compatibility of the biological motor with the tissue that they targeted. For instance, the inherent affinity of macrophages for penetrating into tumors can be helpful in designing macrophage‐based MMBs with the ability to penetrate deep in tumors.^[^ ^138^, ^203^ ^]^ Likewise, the ability of spermbots to deeply penetrate through the cervix and uterus can be leveraged for designing effective niche‐specific bots.^[^ ^43^ ^]^

### 4.3 Optimizing Propulsion Drive at Low Reynolds Numbers

Understanding the prevailing fluid mechanics in the niche of interest is important for designing effective miniaturized bots. Developing MMBs and MNBs that demonstrated a stable motion in low Reynold numbers (Re) regime (Re*≪*1), which are created by intrinsically viscoelastic bacterial biofilms,^[^ ^204^ ^]^ is a challenging mission. Proper design could help bots overcome the dominated viscous forces in such matrices. There is evidence in the literature that designing appendage‐like structures on MMBs, inspired by flagellated microorganisms, could help them achieve back‐and‐forth flagellated propulsions, resulting in oar‐like beats and net displacement.^[^ ^205^, ^206^ ^]^ Creating scallop‐inspired reciprocal movement by periodic body‐shape transformations in magnetic MMBs is another promising strategy for the movement of bots in non‐Newtonian fluids with low Re.^[^ ^207^ ^]^ Artificial intelligence, particularly reinforcement learning, is another tool that has been employed to train MMBs by exploration and exploitation algorithms to generate mechanical net displacement at low Re.^[^ ^208^ ^]^ Utilizing machine learning is a promising trend that is expected to sustain and improve the design and optimization process of MMBs and MNBs.

## 5 Future Perspective on Utilizing Micro‐ and Nano‐Bots for Infection Management

The use of miniaturized robots for infection management is highly promising, but the field is still in its infancy. In this section, we have outlined the trends that we expect to continue shaping the field. These trends include multi‐drive systems integrating various propulsion mechanisms,^[^ ^21^, ^170^, ^209^ ^]^ bioinspired bots that mimic the natural motile behaviors of specialized cells and organisms,^[^ ^210^ ^]^ and applying of artificial intelligence (AI) to train the next generation of autonomous smart MMBs and MNBs.^[^ ^66^ ^]^ We will also discuss the commercialization process of MMBs and MNBs and the challenges in translating from bench to clinic.

### 5.1 Developing Multi‐Drive Micro‐ and Nano‐Bots

To overcome the limitations of each specific propulsion mechanism and build on the advantages of each, researchers have explored integrating different propulsion mechanisms to develop multi‐drive bots (Figure 5b). A notable example is magnetotactic bacteria‐based microbots. Magnetotactic bacteria, discovered in the 1970s, are bacterial species that can naturally orient themselves along the magnetic field of the earth.^[^ ^211^ ^]^ They navigate using the earth's magnetic field as well as their flagella, and thus magnetotactic‐bacteria‐based microbots can be considered as a natural hybrid, a biological‐physical (bacteriobot‐magnetic) MMB.^[^ ^170^, ^177^, ^212^ ^]^ The first report of a magnetotactic‐bacteria‐based MMBs dates back to 2006.^[^ ^213^ ^]^ Since then, numerous studies have focused on developing multi‐drive bots as listed in **Table** **3**. For instance, a bubble‐propulsion – magnetic MMBs, composed of tubular mesoporous silica coated with MnO_2_ and Fe_3_O_4_ nanoparticles was developed and used for mechanical biofilm disruption. The authors reported high‐speed movement and precise navigation, which are essential features for the treatment of deep‐severe infection sites.^[^ ^214^ ^]^ Another report revealed that merging chemical and biological propulsion with self‐diffusiophoretic – macrophage‐based MMBs enables immune evasion immune barriers, speed control, and autonomous adaptation to changes in the biological environment.^[^ ^139^ ^]^

**Table 3:** Advantages of some multi‐drive MMBs and MNBs for infection management.

Type of the robot	Mechanisms of propulsion	Advantages	Applications	Refs.
MNBs and MMBs	Biological and Physical – Bacteriobot and Magnetic (Magnetotactic Bacteria)	Straightforward synthesis, High movement speed, High biocompatibility, Tracking and monitoring visibility	Targeting, killing, and separating *S. aureus* bacteria, Bacterial biofilm eradication	[170, 177, 212, 230]
MNBs and MMBs	Chemical and Physical – Bubble propulsion and Magnetic	High biocompatibility, Precise controllability, High movement speed	Selective capture of Gram‐positive bacteria, Bacterial biofilm eradication	[214, 231]
MNBs	Chemical and Physical – Self‐diffusiophoretic and Optical	Low cost of preparation, Precise controllability, Scalable synthesis, Synergistic antibacterial activity	Bacterial biofilm eradication	[209]
MMBs	Physical and Physical –Optical and Magnetic	Precise controllability, High movement speed, Flexible movement in multiple directions	Modulation of gut bacteria	[232]
MMBs	Biological and Physical –Algae‐based bot and Magnetic	Tracking and monitoring visibility, Low cost of preparation, Precise controllability	Precise photothermal therapy for the treatment of bacterial infection, Infection diagnosis	[21]
MMBs	Biological and Chemical –Macrophage‐based bot and Self‐ diffusiophoretic	Deep penetration, High biocompatibility, Surpassing form multiple biological barriers	Precision treatment of gastrointestinal inflammation	[139]

### 5.2 Designing Bioinspired Micro‐ and Nano‐Bots

**Figure 6:**
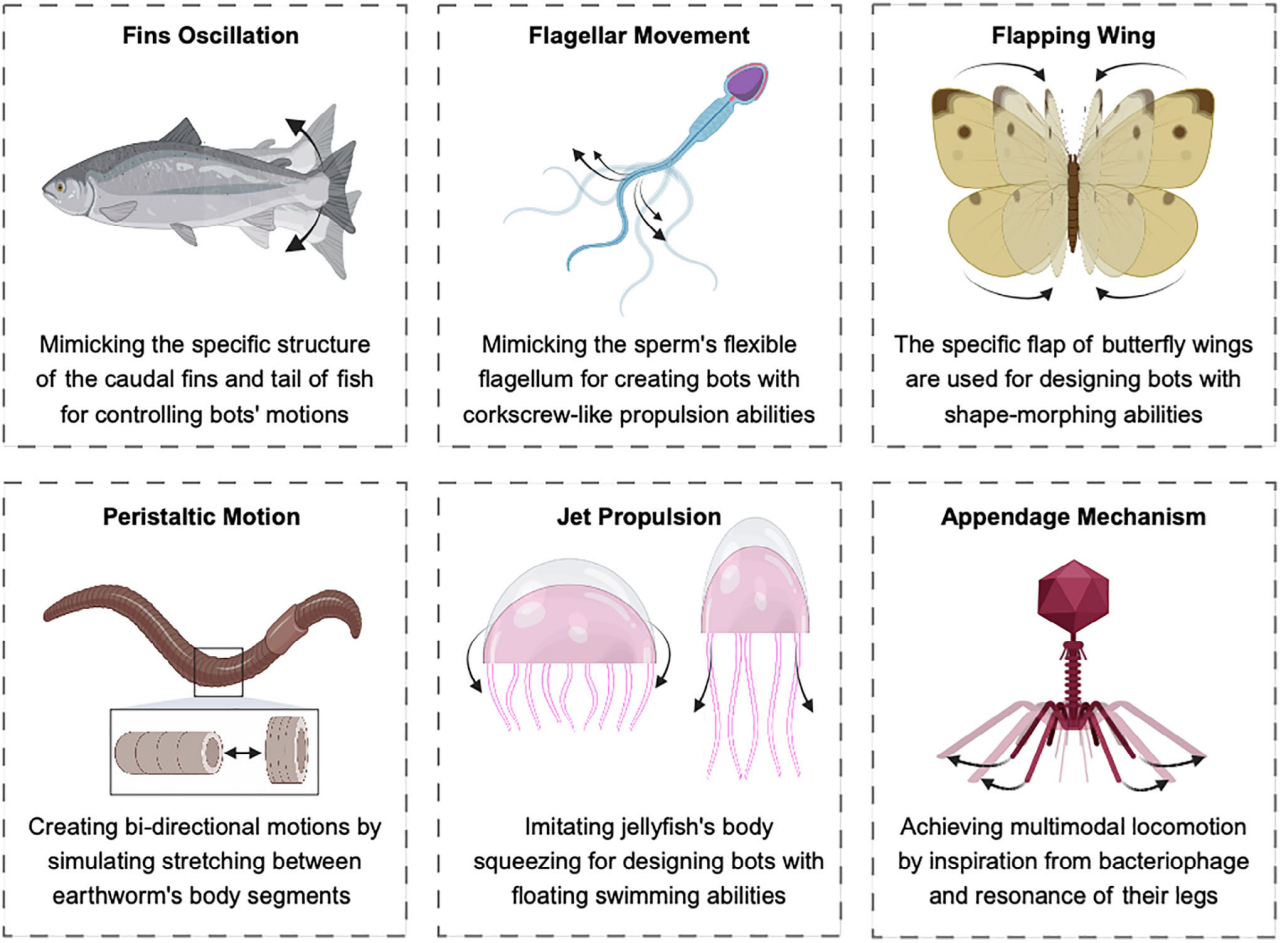
Perspectives on the development of novel MMBs and MNBs based on bioinspiration from natural motile cells and organisms regarding their propulsion mechanisms. These motion mechanisms can be modelled from the principles seen in fish and their fin oscillation, bacteria or sperms and their flagellar movements, butterflies with their flapping wings, earthworms and their peristaltic motion, the jet propulsions of jellyfish, and the multimodal locomotion of bacteriophages.

Motile organisms have evolved to navigate complex environments by responding to chemical, physical, and biological stimuli. By drawing inspiration from the form, bioinspired and biomimetic MMBs and MNBs can move toward mimicking function of motile organisms. As shown in **Figure** **6**, the active mechanics and multiphasic structures of macro, micro, and nanoscale motile cells and organisms can be a rich source of inspiration for developing novel bots. The shape‐changing features of sea creatures, including fish, jellyfish, and crabs, provide key evolutionary design inspirations. Inspired by crabs and fish, environmentally adaptive magnetic MMBs were via developed a 4D printing method to deliver the anticancer drug to tumor cells. These shape‐morphing MMBs composed of pH‐responsive polymer effectively emulated the switchable configuration of the fish fins and crab claws. The deformation also serves to release loaded cargo with high temporal and spatial resolution.^[^ ^34^ ^]^ Terrestrial organisms such as earthworms and butterflies also serve as sources of inspiration for their peristaltic locomotion and aerodynamic performance.^[^ ^215^, ^216^ ^]^

On a smaller scale, the simple and functional form of sperm cells and bacteria, which enable them to swim in complex biological media in swarms and respond to environmental conditions, has long been a source of inspiration in designing MMBs and MNBs.^[^ ^210^ ^]^ Zhang et al. designed sperm‐inspired optical MMBs, consisting of a cylindrical head and a helical tail, that demonstrated linear and rotary motions owing to the asymmetric expansion and shrinkage of their coiled tail under light stimulation.^[^ ^217^ ^]^ In another study, bacteria‐inspired acoustic‐magnetic MMBs showed outstanding upstream motility due to their rheotaxis behavior that enabled switchable orientation to adapt to the gradient of a shear flow.^[^ ^218^ ^]^

Viruses, although nonmotile, have also served as a source of inspiration. Wang et al. designed bacteriophage‐inspired electrical MMBs that were composed of a triangular core that contained piezoelectric elements and resonant legs attached on bases, resembling the capsid and tails of the T‐bacteriophages. This design enables multimodal locomotion, namely climbing and flying movements, with low operation voltage.^[^ ^219^ ^]^

### 5.3 AI‐Driven Micro‐ and Nano‐Bots

The unprecedented advances in AI over the past decade have enabled real‐time data processing and adaptive learning, leading to the emergence of smart MMBs and MNBs trained by AI systems.^[^ ^220^ ^]^ Optimizing navigation parameters and overall efficiency requires examining MMBs and MNBs in various biological media, thus resulting in large datasets that could serve as a starting point for training AI algorithms. Smart bots utilize machine learning algorithms, which are provided by these existing datasets, for generating autonomous optimal decisions under various application scenarios. For example, Yang et al. designed environment‐adaptive magnetic MMB swarms with autonomous navigation that control their shape, orientation, and motion using deep learning. By implementing autonomous trajectory planning algorithms, which depend on environmental morphologies, MMB navigator systems demonstrated intelligent adjustment of motion and configuration patterns based on environmental conditions.^[^ ^221^ ^]^ The results of another study demonstrated the application of reinforcement learning techniques, as one of the main subdivisions of AI, in training acoustic MMBs for autonomous navigation in fluids.^[^ ^222^ ^]^

In addition to applications of AI in training MMBs and MNBs for navigation and locomotion, AI systems can be involved in optimizing other design parameters. Zeng et al. employed machine learning methods to predict the efficiency and speed of chemical MNBs applied for the removal of organic pollutants from water. This study revealed that applying machine learning tools provided the opportunity to create diverse models of MNBs with various shapes, sizes, and materials and computing their predicted performance in an accurate and fast manner.^[^ ^223^ ^]^

### 5.4 Bench‐to‐Bedside Translation

Translating new technologies from the lab to the clinic demands extensive research and costly development to standardize methodologies for analyzing their clinical efficacy. Although MMBs and MNBs can be a game‐changers for infection management, their commercialization path is challenging and involves laborious and costly preclinical testing. The commercialization process of MMBs and MNBs for other bio‐applications such as microbiopsy and microsurgery is also challenging with rigorous testing requirements. In the United States, MMBs and MNBs must generally meet the certification standards of the FDA (Food and Drug Administration) certification standard for category III medical devices.^[^ ^224^ ^]^ This class consists of high‐risk devices that are subject to stringent regulatory requirements. Therefore, the time requirement and final costs for receiving regulatory approval are relatively high.^[^ ^225^ ^]^ Unfortunately, there are currently no commercialized MMBs or MNBs products on the market. However, startup companies have made substantial investments to develop MMBs and MNBs as the next‐generation healthcare products. For example, Bionaut, a leading startup focused on designing magnetic MMBs for therapeutics delivery, has raised over US 70 million to take its approach into phase 1 trials.^[^ ^226^ ^]^ Another example is LIBERTY, which specializes in developing endovascular surgical MMBs and has recently obtained FDA approval to conduct a human clinical trial.^[^ ^227^ ^]^ Finally, it should be noted that according to the application scenario for a MMB or MNB, e.g., administration routes and propulsion mechanisms, establishing a holistic standard for evaluating biocompatibility that ensures short‐ and long‐term safety must be a priority. Formulating standards for commercialization of the first MMBs or MNBs products will pave the way for the fast emergence of other miniature medical robots for a wide range of medical applications. This trend can be expected based on observations in other fields, such as the prompt development of mRNA therapeutics after the commercialization of the Pfizer/BioNTech and Moderna mRNA COVID‐19 vaccines.

## 6 Concluding Remarks

Infectious diseases are an expanding threat to global health and MMBs and MNBs have the potential to be an effective tool for infection prevention, treatment, and diagnosis. To develop these bots as a definitive solution for infection management, it is important to clarify the design principles, e.g., propulsion mechanisms, cargo loading/delivery, size and geometry, etc., in the context of the intended application. MMBs and MNBs with physical propulsion mechanisms, including magnetic, electrical, optical, or acoustic forces, offer fuel‐free and controllable movement that allows them to access hard‐to‐reach sites of the body. However, their navigation could be challenging due to the complexity of the external control systems. In contrast, chemical and biohybrid MMBs and MNBs, which exhibit autonomous motion without the need for specialized infrastructure, are limited by short lifetimes and low controllability, making them less suitable for use in complex biological media, such as deep tissue infections.

With carefully tailored actuation methods, MMBs and MNBs can pave the way for infection management at different stages and transform the landscape of infection prevention, treatment, and monitoring. Developing MMBs and MNBs as minimally invasive surgeons enables surgery at the single‐cell level, reducing the need for large incisions and minimizing post‐surgical infection risks. Furthermore, MMBs and MNBs hold exceptional potential for designing highly sensitive and rapid biosensors that integrate with various transduction mechanisms, enabling early detection of infectious agents and thus decreasing healthcare costs. By utilizing these bots as contrast agent carriers, real‐time monitoring of infected tissues will be more accessible by a potential decrease in cost. Besides, imaging can provide detailed and immediate information about infected patients, which is important for designing personalized treatment plans. Most importantly, MMBs and MNBs hold promise for revolutionizing infection treatment methods through targeted antimicrobial delivery and effective and rapid biofilm eradication.

The outstanding challenge in the field is navigating these bots through dynamic biological media and evading immune clearance, although mitigating strategies such as the application of camouflage coatings, optimization of geometrical dimensions, and tailoring propulsion mechanisms are all promising. Future advancements are expected to continue with a focus on multi‐drive, bioinspired, and AI‐controlled MMBs and MNBs systems. Finally, we believe that the commercialization of the first MMBs or MNBs products will generate the momentum needed for establishing critical safety standards and further streamlining the regulatory process.

## Conflict of Interest

The authors declare no conflict of interest.
